# Neutralization of Interleukin-1β following Diffuse Traumatic Brain Injury in the Mouse Attenuates the Loss of Mature Oligodendrocytes

**DOI:** 10.1089/neu.2018.5660

**Published:** 2018-11-12

**Authors:** Johanna Flygt, Karsten Ruscher, Amanda Norberg, Anis Mir, Hermann Gram, Fredrik Clausen, Niklas Marklund

**Affiliations:** ^1^ Department of Neuroscience, Section of Neurosurgery, Uppsala University, Uppsala, Sweden.; ^2^ Novartis Institutes of Biomedical Research, Basel, Switzerland.; ^3^ Lund University, Skane University Hospital, Department of Clinical Sciences Lund, Neurosurgery, Lund, Sweden.

**Keywords:** fluid percussion injury, inflammation, IL-1β, microglia, oligodendrocyte, oligodendrocyte progenitor cells, Olig2, traumatic brain injury

## Abstract

Traumatic brain injury (TBI) commonly results in injury to the components of the white matter tracts, causing post-injury cognitive deficits. The myelin-producing oligodendrocytes (OLs) are vulnerable to TBI, although may potentially be replaced by proliferating oligodendrocyte progenitor cells (OPCs). The cytokine interleukin-1β (IL-1β) is a key mediator of the complex inflammatory response, and when neutralized in experimental TBI, behavioral outcome was improved. To evaluate the role of IL-1β on oligodendrocyte cell death and OPC proliferation, 116 adult male mice subjected to sham injury or the central fluid percussion injury (cFPI) model of traumatic axonal injury, were analyzed at two, seven, and 14 days post-injury. At 30 min post-injury, mice were randomly administered an IL-1β neutralizing or a control antibody. OPC proliferation (5-ethynyl 2′- deoxyuridine (EdU)/Olig2 co-labeling) and mature oligodendrocyte cell loss was evaluated in injured white matter tracts. Microglia/macrophages immunohistochemistry and ramification using Sholl analysis were also evaluated. Neutralizing IL-1β resulted in attenuated cell death, indicated by cleaved caspase-3 expression, and attenuated loss of mature OLs from two to seven days post-injury in brain-injured animals. IL-1β neutralization also attenuated the early, two day post-injury increase of microglia/macrophage immunoreactivity and altered their ramification. The proliferation of OPCs in brain-injured animals was not altered, however. Our data suggest that IL-1β is involved in the TBI-induced loss of OLs and early microglia/macrophage activation, although not the OPC proliferation. Attenuated oligodendrocyte cell loss may contribute to the improved behavioral outcome observed by IL-1β neutralization in this mouse model of diffuse TBI.

## Introduction

Traumatic brain injury (TBI) is a complex disease induced by a broad spectrum of injury mechanisms that include a robust inflammatory response with both beneficial and deleterious effects on patient outcome.^1^ The pathological features of TBI also include impaired brain network function because of traumatic axonal injury (TAI), the experimental counterpart of diffuse axonal injury (DAI), and concomitant injury to other components of the white matter tracts resulting in persisting motor, sensory, and cognitive disabilities.^2–5^ Oligodendrocytes (OLs) are the primary cells responsible for producing and maintaining the normal myelin sheath.^6^ OLs are vulnerable to inflammation, oxidative stress, and excitotoxicity, all present after TBI.^7–9^ Conversely, oligodendrocyte death has been observed after both experimental and human TBI.^10–12^

The endogenous regeneration of OLs by proliferating OL progenitor cells (OPCs) was observed in several models of focal and diffuse TBI.^13–16^ The stimulating and/or suppressive signals for OPC proliferation and myelin remodeling after TBI have not been established. OPCs may be inhibited, promoted, or differentiated by several secreted signaling molecules, however, as well as by several cell-adhesion and extracellular matrix molecules.^17^ In addition, the inflammatory reaction elicited by TBI can both stimulate and inhibit OPC proliferation via different chemokines and cytokines.^18^

One key player is the pro-inflammatory cytokine interleukin-1β (IL-1β), increased after both experimental and human TBI.^19–21^ IL-1β is produced by activated microglia, astroglia, endothelial cells, and recruited leukocytes^22^ and may have a dual role in OPC proliferation and myelin remodeling because it may be cytotoxic to mature OLs.^9,23,24^ It can also stimulate the production of growth factors known to induce the proliferation of OPCs. By using an antagonist to the IL-1 receptor, morphological and histological outcome was improved after experimental TBI.^23,25,26^ In a series of experiments from our group, neutralization of IL-1β attenuated the inflammatory response, cerebral edema, and tissue loss and improved cognitive outcome in a focal TBI model in mice.^27,28^ Recently, neutralization of IL-1β normalized the complex behavioral changes observed in a diffuse TBI model, the central fluid percussion model, in mice.^29^ The mechanisms explaining the behavioral improvements observed by IL-1β neutralization have not been established.

In this report, we hypothesized that neutralization of IL-1β may influence OL cell death, increase OPC proliferation, and attenuate microglial/macrophage activation after diffuse TBI in the mouse.

## Methods

### Animals

One hundred and sixteen adult male C57BL/6 mice (pre-injury weight 25 ± 1.7 g; Taconic, Denmark) were housed in groups of six per cage, with access to food and water *ad libitum,* on a 12 h light/dark cycle. The animals were housed in the animal care facility for a minimum of seven days before any experiments. All experiments were approved by the Uppsala County Animal Ethics board and followed the regulations of the Swedish Animal Welfare Agency.

### Surgical procedure

Mice were subjected randomly to sham injury (*n* = 38) or central fluid percussion brain injury (cFPI; *n* = 78). Based on previous results in TBI models showing early oligodendrocyte death and transient OPC proliferation with a peak at seven days post-injury,^10,16^ three survival end-points were used; two days post-injury (dpi), seven dpi, and 14 dpi. The cFPI protocol was adapted to mice and used in the present report as described previously.^16,30^ Anesthesia (4% isoflurane in air) was induced in a chamber, and the animals were then moved to a stereotaxic frame where anesthesia was maintained through a nosecone delivering isoflurane 1.2% and N_2_O/O_2_ 70/30% and spontaneous breathing. The scalp was shaved and cleaned with ethanol, and bupivacaine 2.5 mg/mL (Marcaine^®^, AstraZeneca, Sweden) was applied subcutaneously. A midline incision was made and the skull exposed.

A 3.0 mm diameter craniotomy was made in the midline between bregma and lambda sutures, carefully leaving the underlying dura intact. A dural breach was an exclusion criterion. A plastic cap was attached over the craniotomy using dental cement (HeraeusKulzer GmbH, Hanau, Germany). Saline was added into the cap, and any signs of saline leakage were noted. If the seal was intact, the saline cap was attached to the Luer-Lock on the fluid percussion device (VCU Biomedical Engineering Facility, Richmond, VA). The injury was produced by releasing the fluid percussion pendulum striking a saline-filled cylinder creating a pressure wave transmitted into the closed cranial cavity. A transducer measured the pressure pulse and the pressure peak noted in pressure by square inch (psi).

Immediately after the injury, an apnea was noted, and when spontaneous breathing resumed, anesthesia was resumed. An apnea >60 sec was used as an exclusion criterion. The cement and cap were removed and the bone flap replaced over the craniotomy and the skin sutured using resorbable sutures. The animal was placed in a separate cage until fully recovered from anesthesia. Animal weight was recorded for a minimum of three days after the injury. If weight loss more than 10% was noted, the animal was sacrificed and excluded from the study. Sham-injured animals were subjected to an identical procedure as the cFPI animals except that the pendulum was not released.

All animals were sacrificed by an intraperitoneal (ip) overdose of sodium pentobarbital (pentobarbital natrium 60 mg/mL, VET ATL, Apoteket, Sweden; 200 mg/kg. Cardiac perfusion was performed using 4% formaldehyde (formaldehyde 4% Fosfatbuffrad, HistoLab Products AB, Gothenburg), and the brains were removed rapidly from the cranium, placed in formaldehyde for 24 h, and then placed in 30% sucrose solution for 72 h until snap frozen in isopentane and stored at −70°C until sectioned.

### EdU and IL-1β neutralizing antibody administration

The IL-1β neutralizing antibody (IL-1β; 300 μg/dose) or control anti-cyclosporinA mlgG2a (CsA; 500 μg/dose), kindly provided by Novartis, Inc., Basel, Switzerland, was administered ip 30 min after sham injury or cFPI for all evaluated groups. The 14 dpi group received a second dose at seven dpi. The CsA control antibody is directed toward unnatural amino acids of a fungal peptide, and no pharmacological activity is expected because there is no cross-reaction with mammalian epitopes. Several previous studies used this treatment regime, suggested by the manufacturer, and observed brain penetration of the antibody and improved behavioral and histological outcome in different TBI models.^27–29^

To study OPC proliferation, 50 mg/kg of 5-ethynyl-2′-deoxyuridine (EdU)^31,32^ was administrated ip after sham injury or cFPI. The two dpi group received one dose of EdU 24 h post-injury. The seven dpi group received six doses of EdU 24 h post-injury until 24 h before sacrifice. The 14 dpi group received six doses of EdU days 8–13 post-injury.

### Tissue preparation and image processing

A sliding microtome (SM 2000R; Leica, Nusstoch, Germany) was used to make 20 μm thick coronal sections. All sections were collected and placed in cryoprotectant buffer (30% glycerin, 30% ethylenglycerol, and 40% 1x phosphate buffered saline [PBS]) and stored at −20°C. Images for quantification were captured using a bright field and fluorescent microscope system, Zeiss Axiovision (Carl Zeiss Inc. Gottingen, Germany). Quantification was made from × 20 images (138.79 μm × 103.99 μm). Co-localization studies (EdU/Olig2/4′,6-diamidino-2-phenylindole [DAPI], cleaved caspase-3/MOG/DAPI) and microglia/macrophage stainings were studied by confocal microscopy (Zeiss LSM 510 laser scanning microscope; Carl Zeiss Inc. Gottingen. Germany), and ZEN 2012 blue edition software (Carl Zeiss Microscopy GmbH, Jena, Germany) was used for image processing.

All quantifications were made by an observer blinded to type of injury and treatment. The number of cleaved caspase-3 positive stainings was quantified from one image (138.79 μm × 103.99 μm) of the corpus callosum from three levels per animal (−1.5, −2.0, and −2.5 mm from bregma). The corpus callosum was outlined using ImageJ (National Institutes of Health, Washington, DC) and then converted to a 32-bit grayscale image and threshold was set. The number of cleaved caspase-3 positive stainings was estimated using the ImageJ software particle analysis.

The number of OLs, defined as labeled by the oligodendrocyte-specific marker CC1, in the corpus callosum and external capsule, −1.5, −2.0, and −2.5 mm form bregma, was quantified using the ImageJ software. One image from the corpus callosum and four images from the external capsule were analyzed from each level by converting the images to 32-bit grayscale images, setting threshold, and measuring the number of cells positive for CC1 staining by particle analysis in ImageJ, as described previously.^13^ The corpus callosum and external capsule were outlined in each image, and only the cells within the outline were included in the analysis. The number of cells in the four images of the external capsule was added together, results presented as cells/mm^2^, and total number of cells from all three levels included.

All cells positive for EdU in the corpus callosum, external capsule, and fimbriae were photographed in three levels, −1.5, −2.0, and −2.5 mm from bregma, for each animal. EdU/DAPI and EdU/Olig2/DAPI positive cells were counted, and results are presented as cells/mm^2^.

*In situ* hybridization was performed to co-localize EdU positive cells with Olig2 ribonucleic acid (RNA) transcripts and the nuclear marker DAPI. Ten RNAOlig2/EdU/DAPI positive cells from the corpus callosum and external capsule at −2.0 mm from bregma were analyzed in × 63 magnification. All Olig2 RNA transcripts, where each dot in the image corresponds to one single RNA transcript, were counted in three animals per group by an observer blinded to the injury and treatment status of the animals. Co-localization of cleaved caspase-3/MOG (myelin-oligodendrocyte-glycoprotein) was also made with *in situ* hybridization to confirm apoptotic OLs.

### Immunohistochemistry

Cleaved caspase-3 staining and staining for CC1 positive mature OLs was used to study OL cell loss. Sections were placed in 1x PBS +0.1% triton and washed 3 × 5 min. The sections were then blocked with 5% normal goat serum in 1x PBS +0.1% triton at room temperature for 1 h. The sections were placed in 0.3% triton in 1x PBS at 80°C for 20 min and then citrate buffer (pH 6.0) for 20 min at 80°C and washed again. The primary antibody (anti-cleaved caspase-3, 1:300, Cell Signaling Technology, Boston, MA) was applied in 1x PBS +0.1% triton in room temperature on a rocking plate overnight. The sections were washed and the secondary antibody was applied for 1 h (1:500, 555 Alexa Fluor Invitrogen Molecular Probes, Eugene; OR). The sections were washed again and the second primary antibody (anti-CC1, 1:300, Abcam, Cambridge, UK) was applied in 1x PBS +0.1% triton in room temperature on a rocking plate overnight. The sections were washed and the second secondary antibody was applied (1:500, 488 Alexa Fluor Invitrogen Molecular Probes, Eugene; OR) for 1 h. After washing the sections, the nuclear marker DAPI was applied for 5 min. The sections were washed and mounted (Everbrite Hardset mounting medium, Biotium, Hayward, CA).

OPC proliferation was studied by EdU labeling using the Click-iT^®^ assay together with immunohistochemistry for Olig2, a transcriptional factor expressed in OLs and up-regulated in OPCs, and the nuclear stain DAPI. The sections were washed in 1x PBS +0.1% triton for 3 × 5 min and blocked with 5% normal goat serum in 1x PBS +0.1% triton at room temperature for 1 h. EdU cells were detected with Click-iT^®^ assay according to manufacturers' protocols; the sections were washed and placed in citrate buffer (pH 6.0) for 15 min at 80°C and washed again. The primary antibody anti-Olig2 (1:500, Millipore, Darmstadt, Germany) in 1x PBS +0.1% triton was applied in room temperature on a rocking plate overnight. The sections were washed and the secondary antibody was applied for (1:500, 555 Alexa Fluor Invitrogen Molecular Probes, Eugene, OR) for 1 h. The nuclear stain DAPI from the Click-iT^®^ kit was applied for 30 min and the sections then were washed and mounted (Everbrite Hardset mounting medium, Biotium, Hayward, CA).

Microglia/macrophages were detected using the ionized calcium binding adaptor molecule 1 (Iba 1) (1:1000, Wako Chemicals, Neuss Germany), an accepted marker for activated microglia/macrophages,^33–35^ and neutrophils by anti-GR-1 (1:200, Bioledgend, San Diego, CA) by washing the sections 3 × 5 min in 1x PBS **+** 0.1% triton. The sections were blocked using 5% normal donkey serum in 1x PBS +0.25% triton at room temperature for 1 h. The primary antibody was applied overnight and the secondary antibody (1:500, Cy3 Alexa Fluor Invitrogen Molecular Probes, Eugene, OR) for 90 min in room temperature and cover-slipped using PVA-DABCO (Saveen-Werner, Malmö, Sweden).

### In situ *RNA hybridization*

RNAScope^®^ technology (Advanced Cell Diagnostics Milan, Italy) was used for *in situ* RNA hybridization according to the manufacturer's protocol. The sections were washed in 1x PBS **+** 0.1% triton for 3 × 5 min and then placed on glass slides and air dried. The slides were incubated in warmed pre-treatment 3 (Advanced Cell Diagnostics Milan, Italy) for 5 min, kept at a light boil, and then placed in distilled water. The slides were then subjected to RNAscope^®^ Multiplex Fluorescent Assay by first incubating the probe (mmOlig2, mmCasp3, or mm MOG) for 2 h at 40°C and then pre-forming the amplification steps according to instructions. The slides used for Olig2/EdU labeling were then blocked with 5% normal goat serum in 1x PBS +0.1% triton at room temperature for 1 h, and the Click-iT^®^ assay (described above) was then performed followed staining with the nuclear marker DAPI for 5 min. The sections were washed and mounted (Everbrite Hardset mounting medium, Biotium, Hayward, CA).

### Analysis of microglial/macrophage reactivity

Sholl analysis was performed to evaluate ramification of microglial/macrophage cells^36^ in the corpus callosum at two, seven, and 14 days after cFPI and sham injury. The number of intersections of a cell with circles of increasing diameters indicates the degree of ramification of the cell. Sections were stained for Iba-1. Z-stacked confocal images were merged into a single plane image using the LSM Image Browser software (Carl Zeiss Inc, Gottingen, Germany). Per picture, three cells per animal were selected randomly, using a circle with a circumference of 100 μm (140/π in diameter). The following selection criteria for analysis were defined: (1) a cell needed to completely fit in the circle; (2) a cell needed to have a visible soma; and (3) a cell should not overlap with other cells. Using ImageJ, pictures were converted to eight-bit files with a set threshold. The center of a cell was defined manually, and Sholl analysis was performed by starting with a circle with a diameter of three pixels and expanding the circles with one pixel each time. Intersections per circle were counted using the ImageJ Sholl analysis plugin up to 60 intersections. Results from each cell per conditions were integrated and analyzed using Microsoft Excel.

Areas of Iba-1 immunoreactivity were also quantified in the corpus callosum and bilateral external capsule after cFPI and sham-injury. Areas have been delineated according to our previous study.^10^ Z-stacked micrographs from coronal brain sections were acquired using a Zeiss LSM510 laser scanning confocal microscope and merged into a single plane image using the LSM Image Browser software. Thereafter, micrographs were converted into binary eight-bit pictures and the area covered by immunoreactivity in the corpus callosum and external capsule was quantified as percentage of total image area using the ImageJ software (NIH, Washington, DC).

### Statistics

The data were analyzed for Gaussian normal distribution using the Shapiro-Wilks test. The data did not meet the assumption of normal distribution, and the Kruskall-Wallis test was used; if *p* ≤ 0.05, the Mann Whitney *U* test was performed. A *p* value ≤0.05 was considered significant. All statistical analysis was performed with IBM SPSS 20 (SPSS IBM, Chicago, IL). Graphs are presented with mean value and standard error of the mean (SEM).

## Results

### Animals

One sham-injured animal died in its home cage, and 15 cFPI animals died at time of impact (one cFPI animal was over-anesthetized, one excluded because of a dural breach, and the remaining animals from long-lasting, terminal apnea), resulting in a cFPI-related mortality rate of 13%. One sham-injured animal had a dural rift at operation and was also excluded from the study. Five animals could not be analyzed because of technical problems during tissue preparation (inadequate perfusion or tissue sectioning). Three animals (one cFPI CsA, one cFPI IL-1β, and one sham CsA) were statistical outliers in the immunohistochemical analyses with data more than two standard deviations from the mean value of the group and were excluded from all statistical analyses.

The total number of included animals in the study was 91. In the two dpi group, the number of included animals per group was Sham CsA (*n* = 4), Sham IL-1β (*n* = 7), cFPI CsA (*n* = 11), and cFPI IL-1β (*n* = 8). In the seven dpi group, the number of included animals per group was Sham CsA (*n* = 6), Sham IL-1β (*n* = 5), cFPI CsA (*n* = 8), and cFPI IL-1β (*n* = 9), and in the 14 dpi group, the number of included animals per group was Sham CsA (*n* = 6), Sham IL-1β (*n* = 5), cFPI CsA (*n* = 11), and cFPI IL-1β (*n* = 11). Post-injury apnea, observed in all brain-injured animals, was 27 ± 15 sec (range 10–50 sec). The cFPI device created a pressure pulse of 19 ± 1.6 psi.

### Oligodendrocyte cell death

**FIG. 1.**
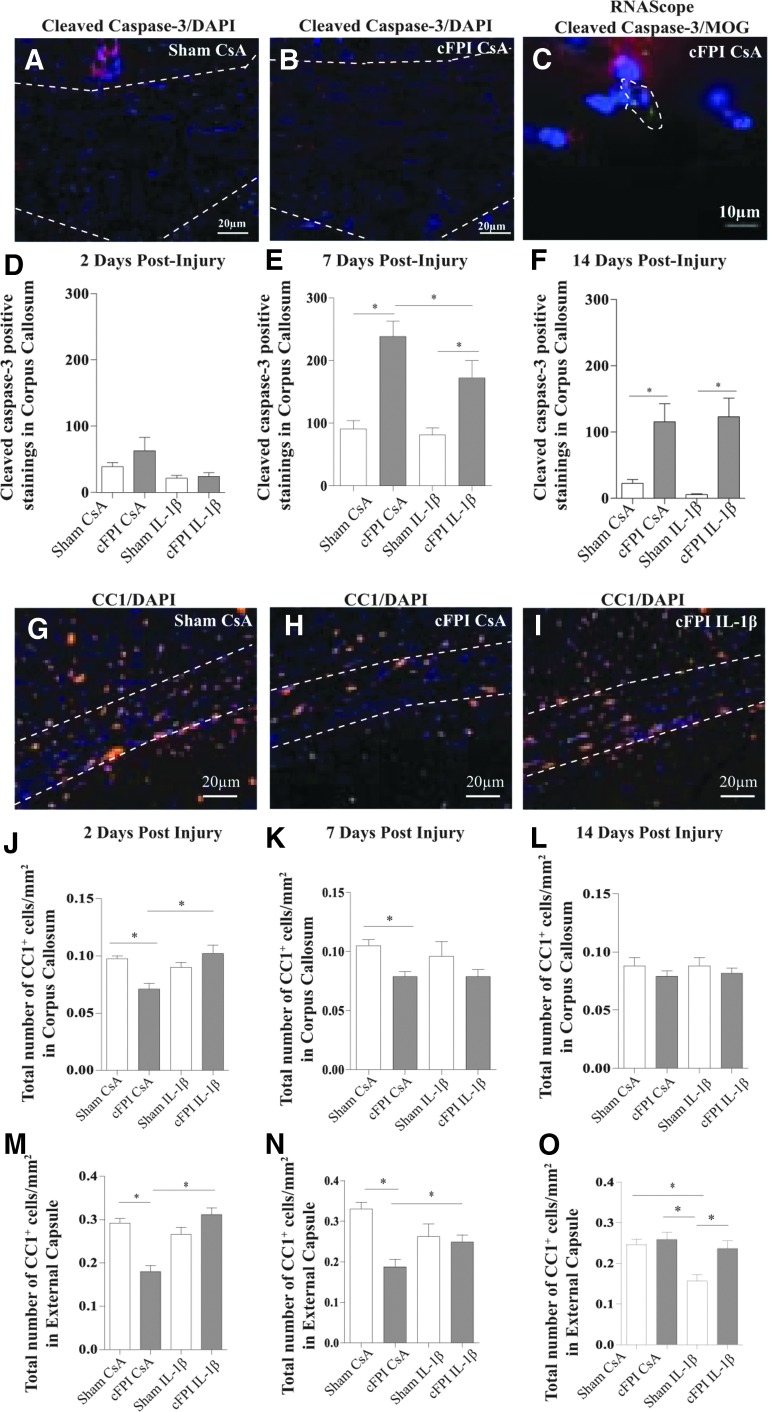
Attenuated oligodendrocyte cell death by neutralization of IL-1β. Compared with sham CsA animals at seven dpi (**A**), the expression of cleaved caspase-3 (red) expression was increased in cFPI CsA animals (**B**). Cell nuclei were defined by DAPI staining (blue). (**C**) Cleaved caspase-3 ribonucleic acid expression (red) was detected in MOG positive OLs (green) co-labeled with DAPI (blue) in cFPI CsA animals at seven dpi. (**D**) At two dpi, cleaved caspase-3 expression was increased in cFPI CsA animals compared with other groups (sham CsA, sham IL-1β, and cFPI IL-1β), and at seven dpi (**E**), this increase was significant (*p ≤ 0.05) in the brain-injured groups (cFPI CsA and cFPI IL-1β) compared with the sham-injured groups (sham CsA and sham IL-1β). Cleaved caspase-3 expression was attenuated in brain-injured animals treated with the IL-1β neutralizing antibody (*p ≤ 0.05). (**F**) At 14 dpi, cleaved caspase-3 expression was increased in both brain-injured groups (cFPI CsA and cFPI IL-1β, *p ≤ 0.05) compared with the sham-injured groups (sham CsA and sham IL-1β). (**G**) Mature CC1-positive OLs (orange) with nuclear stain DAPI (blue) in sham CsA animals at seven dpi were found throughout the external capsule (marked white area). (**H**) In cFPI CsA animals (example from seven dpi), there was a reduced number of mature OLs, which was attenuated by the IL-1β neutralization (**I**; cFPI IL-1β). (**J**) At two dpi, a loss of mature OLs was detected in the corpus callosum (*p ≤ 0.05) and external capsule (**M**; *p ≤ 0.05) of cFPI CsA animals compared with the sham CsA group. The cFPI-induced loss of mature OLs was attenuated by the IL-1β neutralization (*p ≤ 0.05). (**K**) At seven dpi, OL loss was still detected in the corpus callosum (*p ≤ 0.05) of the cFPI CsA group compared with the sham CsA group. (**N**) In the external capsule, OL loss (cFPI CsA) was still attenuated by IL-1β neutralization in the cFPI IL-1β group (*p ≤ 0.05). (**L**) At 14 dpi, no difference was detected in the number of OLs among the treatment groups in the corpus callosum. In the external capsule (**O**), however, the sham IL-1β group showed a loss of mature OL when compared with other groups (sham CsA, sham IL-1β, and cFPI IL-1β; *p ≤ 0.05). Graphs are presented as mean and standard error of the mean. OL, oligodendrocyte; cFPI, central fluid percussion injury; dpi, days post-injury; CsA, inactive control antibody against cyclosporin A, IL-1β, interleukin 1 beta; MOG, myelin-oligodendrocyte-protein; DAPI, 4',6-diamidino-2-phenylindole.

The distribution of cleaved caspase-3 positive stainings in cFPI animals was seen in the corpus callosum, hippocampus, and external capsule and is schematically presented in Supplementary Figure 1; see online supplementary material at ftp.liebertpub.com). The sham-injured animals displayed minor cleaved caspase-3 expression (Fig. 1 A). Cleaved caspase-3 expression was non-significantly higher in the corpus callosum at two dpi (Fig. 1 D) in the cFPI CsA group, and was significantly increased at seven dpi compared with the other groups (Fig.1 B and E). In brain-injured animals, the cleaved caspase-3 expression was reduced in the IL-1β neutralized group (Fig. 1 E). At 14 dpi, both brain-injured groups showed significantly increased cleaved caspase-3 expression compared with the sham-injured groups (Fig. 1 F), without difference between the IL-1β and control-antibody treated brain-injured groups. Only minor cleaved caspase-3 staining was observed in the external capsule and therefore not included in the analysis.

*In situ* hybridization of caspase-3 confirmed an increased expression of caspase-3 RNA in the corpus callosum of brain-injured animals compared with controls (data not shown). Co-localization of cleaved caspase-3 and mature OLs in the corpus callosum was confirmed by *in situ* hybridization of caspase-3 and MOG RNA transcripts (shown for cFPI CsA, seven dpi, Fig.1 C).

The number of mature OLs was quantified in the corpus callosum and external capsule at three levels per animal, using the mature OL marker CC1. Results are presented as CC1-positive cells/mm^2^. Several of the images from the external capsule frequently displayed very few CC1 positive OLs in the cFPI CsA group, as low as 12 cells per image. There was no difference in the number of CC1 positive OLs between the cFPI IL-1β and the Sham IL-1β groups in any of the investigated areas or levels at two and seven dpi. The cFPI CsA group had a decreased number of CC1 positive OLs in the external capsule and corpus callosum at day two and seven post-injury (Fig. 1G, 1H). Treatment with the IL-1β neutralizing antibody in cFPI animals resulted in an attenuated loss of mature OLs (Fig. 1I) compared with cFPI CsA animals.

At two dpi, cFPI resulted in a loss of OLs in the corpus callosum and external capsule of cFPI CsA animals compared with the sham CsA group. The number of OLs in cFPI IL-1β animals was similar as in sham-injured controls. The reduction of OLs was significantly attenuated by IL-1β neutralization in the brain-injured groups (Fig. 1J, 1M; *p ≤ 0.05). At seven dpi, the number of OLs in the corpus callosum and external capsule was still reduced in the cFPI CsA group compared with sham CsA animals (Fig. 1K, 1N). In the cFPI IL-1β group, the loss of mature OLs was attenuated in the external capsule (Fig. 1N; *p ≤ 0.05) although not in the corpus callosum when compared with the cFPI CsA group (Fig. 1K). At 14 dpi, no difference was detected in the corpus callosum between any of the included groups (Fig. 1L). The sham-injured group treated with antibodies against IL-1β, however, showed a significantly reduced number of OLs in the external capsule at this time-point (Fig. 1O; *p ≤ 0.05).

### Oligodendrocyte progenitor cell proliferation

OPC proliferation was studied in three sections from each animal, and the number of cells was combined and presented as cells/mm^2^. Three regions of interest were investigated: the corpus callosum, external capsule, and fimbriae.

Proliferating cells were present in all regions of interest in sham-injured animals. Co-labeled EdU/DAPI positive cells were increased in the white matter (corpus callosum, external capsule, and fimbriae included) of the brain-injured groups compared with the sham-injured groups at 7 dpi, although not at 2 or 14 dpi (Supplementary Fig. 2; see online supplementary material at ftp.liebertpub.com).

**FIG. 2.**
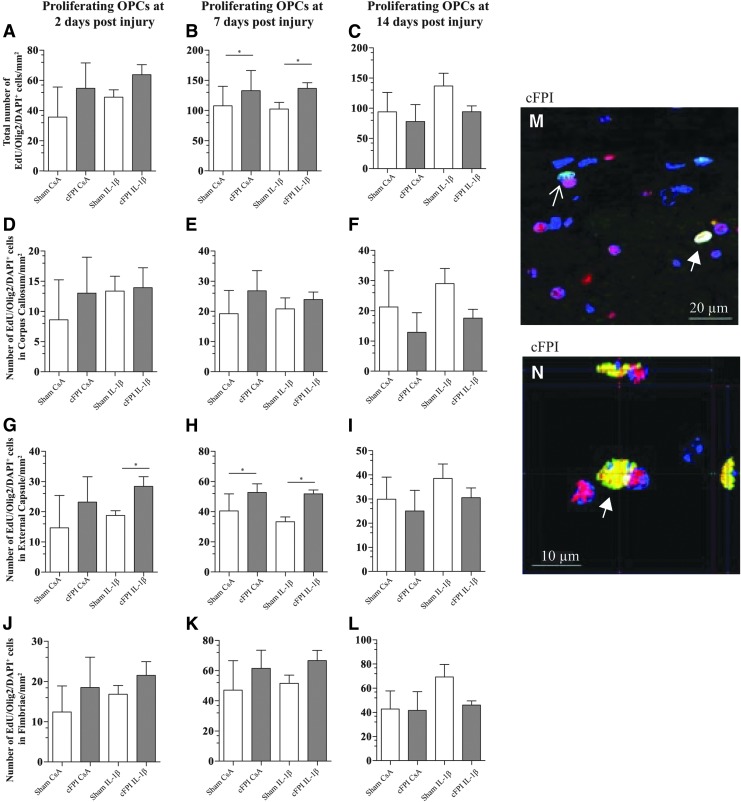
Neutralization of IL-1β does not alter the number of proliferating OPCs. Proliferating OPCs were quantified in the corpus callosum, external capsule, and fimbriae. (**A**) At two dpi, the total number of proliferating OPCs was higher in the brain-injured groups (cFPI CsA and cFPI IL-1β) compared with the sham-injured groups (sham CsA and sham IL-1β); this increase was significant at seven dpi (**B**; *p ≤ 0.05). (**C**) No difference in OPC proliferation was observed among the groups at 14 dpi. There was no difference in OPC proliferation in the corpus callosum at any time point or among any of the groups (**D–F**). In the external capsule, increased OPC proliferation was seen at two (**G**; *p ≤ 0.05) and seven (**H**; *p ≤ 0.05) dpi in the cFPI IL-1β group compared with the sham IL-1β group, and between both brain-injured groups and sham-injured groups, respectively. This increase in OPC proliferation was not sustained at14 dpi (**I**). In the fimbriae, there was no difference in OPC proliferation among any of the groups or time points (**J–L**). For all evaluated time points and brain regions, neutralization of IL-1β did not alter OPC proliferation (A–L). OPC proliferation was detected by co-localization of Olig2 (red)/EdU(green)/DAPI(blue)-positive cells, confirmed by confocal microscopy (M and N). White arrow points to triple positive cells and open arrows to EdU/DAPI positive cells. Graphs are presented as mean and standard error of the mean. OPC, oligodendrocyte progenitor cell; cFPI, central fluid percussion injury; dpi, days post-injury; CsA, inactive control antibody against cyclosporin A; IL-1β, interleukin 1 beta; DAPI, 4',6-diamidino-2-phenylindole.

Approximately half of all proliferating cells were positive for the OPC marker Olig2. OPC proliferation was quantified by triple positive EdU/Olig2/DAPI positive cells and confirmed by confocal microscopy (Fig. 2M, 2N). The total number of EdU/Olig2/DAPI positive cells was increased in the brain-injured groups compared with the sham-injured groups (Fig. 2A–C) at seven although not at two or 14 dpi. No increased proliferation was detected in the corpus callosum and fimbriae at two, seven, and 14 dpi (Fig. 2D, 2J, 2E, 2K, 2F, and 2L). At two dpi, an increased proliferation was detected in the external capsule in the cFPI IL-1β group when compared with the sham IL-1β group (Fig. 2G). At seven dpi, brain-injured groups had in increased OPC proliferation compared with the sham-injured groups (Fig. 2H), although no difference between the groups was observed in the external capsule at 14 dpi (Fig. 2I). In the brain-injured groups, neutralization of IL-1β neither did result in an altered OPC proliferation in any evaluated white matter region nor at any evaluated time-points.

**FIG. 3.**
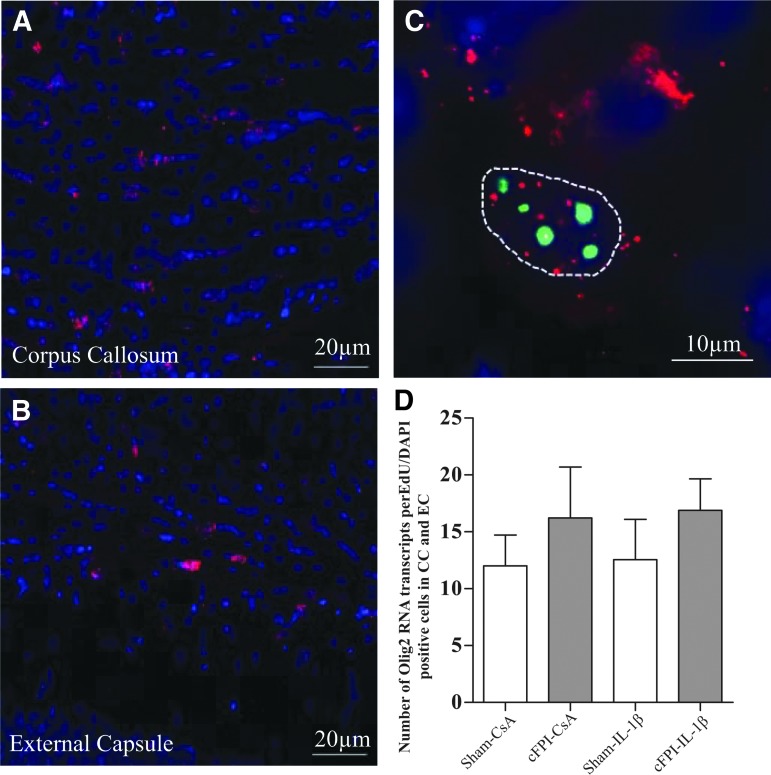
RNA Olig2 expression. The number of Olig2 RNA transcripts (red) was quantified from 10 cells/animal in the corpus callosum (**A**) and external capsule (**B**) in EdU (green), DAPI (blue) positive cells. Each cell was outlined, and all Olig2 RNA transcripts were counted within the outline (**C**). No difference was observed in the number of transcripts between the groups (**D**). Graphs are presented as means and standard error of the mean. RNA, ribonucleic acid; DAPI, 4',6-diamidino-2-phenylindole.

*In situ* hybridization of Olig2 RNA transcripts (Fig. 3A–C) did not detect a difference in the number of transcripts per EdU/DAPI positive cell among the included groups (Fig. 3D).

### Microglia/macrophage morphology and immunoreactivity

**FIG. 4.**
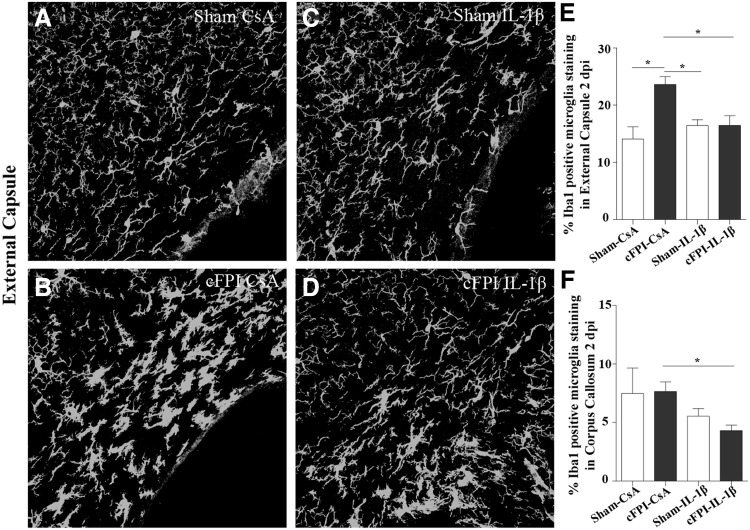
Early microglia/macrophage immunoreactivity is reduced by IL-1β neutralization. At two dpi, Iba-1 positive microglia/macrophage processes appeared thin and ramified in the sham CsA group (**A**) compared with thicker processes in the cFPI CsA animals (**B**) in the external capsule. When the IL-1β neutralized groups are compared (sham IL-1β; **C** and cFPI IL-1β; **D**), no difference was observed in microglia/macrophage processes in the external capsule at this time point. The Iba-1 immunoreactivity was quantified in the external capsule and found increased in cFPI CsA animals compared with all other groups (sham CsA, sham IL-1β, and cFPI IL-1β; **E** *p ≤ 0.05). In the corpus callosum, the Iba-1 immunoreactivity was reduced by IL-1β neutralization in the brain-injured groups (cFPI CsA vs. cFPI IL-1β) at two dpi (**F**; *p ≤ 0.05). Graphs are presented as mean and standard error of the mean. cFPI, central fluid percussion injury; dpi, days post-injury, CsA, inactive control antibody against cyclosporin A, IL-1β, interleukin 1 beta.

Microglia/macrophages were identified by Iba-1 antibody in both its ramified and amoeboid form^33^ (Supplementary Fig. 3; see online supplementary material at ftp.liebertpub.com). At two dpi, branches of Iba-1 positive microglia/macrophages in the corpus callosum and external capsule of cFPI-CsA animals appeared thicker when compared with sham-injured animals (Fig. 4A, 4B). These morphological changes were not observed in Iba-1 positive microglia/macrophages of IL-1β treated mice (Fig. 4C, 4D). At later time points (seven and 14 dpi), these differences were no longer observed, and similar morphologies of Iba-1 positive cells were found among all treatment groups (data not shown). These observations were confirmed by quantification of areas covered by Iba-1 immunoreactivity in the external capsule and corpus callosum.

As shown in Figures 4E and 4F, a significant increase of Iba-1 immunoreactivity was found in mice subjected to cFPI CsA two dpi while mice treated with the neutralizing IL-1β antibody displayed levels similar to those of sham-injured animals (sham CsA: 7.05 ± 0.72%, sham- IL-1β: 8.21 ± 0.45%, cFPI CsA: 12.04 ± 0.63%, cFPI- IL-1β: 8.23 ± 0.99%). No treatment effects were found in sham-injured animals. After seven and 14 dpi, no significant differences in Iba-1 immunoreactivity were observed between the experimental groups. These results show a subacute microglial/macrophage response after cFPI ameliorated by treatment using an IL-1β neutralizing antibody. To exclude bacterial infection, stainings were made for the neutrophil marker GR-1 on 14 dpi section. No neutrophils were detected (results not shown).

**FIG. 5.**
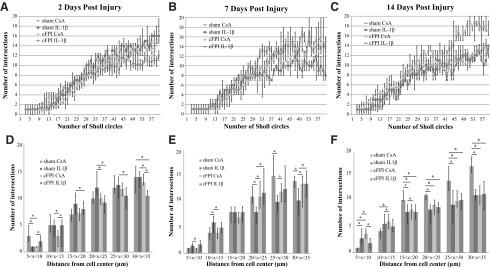
Microglia/macrophage morphology is altered by IL-1β neutralization. Sholl analysis showed changes in microglia/macrophage morphology between the different treatment groups (sham CsA, Sham IL-1β, cFPI CsA, and cFPI IL-1β) and time points (2, 7, and 14 dpi). (**A**) At two dpi, microglia/macrophages of cFPI IL-1β animals appeared more active compared with other groups with a lower number of intersections and increasing number of Sholl circles. (**B**) At seven dpi, microglia/macrophage of sham IL-1β animals were more active compared with other groups. At 14 dpi (**C**), microglia/macrophages of sham IL-1β and brain-injured animals showed the same level of activation as brain-injured animals, and microglia/macrophages of sham CsA animals were more ramified indicated by an increased number of intersections with increasing number of Sholl circles. When intersections at different intervals from the cell center were analyzed, microglia/macrophages of sham IL-1β animals appeared less active at two dpi (**D**), although became more active at seven dpi (E) compared with microglia/macrophages of other groups. At 14 dpi (**F**), microglia/macrophages of sham IL-1β animals and microglia of brain-injured animals displayed the same level of activation. Thus, the microglia/macrophages of sham CsA animals were more ramified. Graphs are presented as mean and standard error of the mean. cFPI, central fluid percussion injury; dpi, days post-injury; CsA, inactive control antibody against cyclosporin A; IL-1β, interleukin 1 beta.

Microglial/macrophage morphology in the corpus callosum was studied using the Sholl analysis protocol.^37^ The results from the analysis are presented in two ways. The number of intersections versus the number of Sholl circles are presented in linear graphs (Fig. 5A–C). This represents increased ramification of the microglia/macrophages with increased distance from the cell soma. Intersections were grouped depending on the distance from the cell center (5–10 μm, 10–15 μm, 15–20 μm, 20–25 μm, 25–30 μm, and 30–35 μm), and the number of intersections at these intervals were analyzed and presented in bar charts (Fig. 5D–5F).

At two dpi, there were few differences noted in the number of intersections between the groups (Fig. 5A). When the interval levels were analyzed, differences in the number of intersections were observed between the sham IL-1β and the two brain-injured groups, where the sham IL-1β group displayed a higher number of intersections (Fig. 5D). At seven dpi, no differences were detected in the number of intersections among the groups (Fig. 5B). Studying the number of intersections in the different intervals, however, the sham IL-1β group had significant less intersections compared with the sham CsA group and the brain-injured groups (Fig. 5E). At 14 dpi, there was a significant increase in the number of intersections in the sham CsA group, predominantly when compared with the cFPI CsA group but also with the sham IL-1β group (Fig. 5C). The sham CsA group also had an increased number of intersections at the last four intervals compared with the sham IL-1β and the cFPI CsA groups. The sham IL-1β group frequently showed decreased number of crossings compared with the cFPI CsA group (Fig. 5F).

## Discussion

In the present study, we hypothesized that an IL-1β neutralizing antibody, previously shown to improve behavioral outcome in experimental TBI,^27–29^ could alter OL cell death, proliferation of OPCs, and microglial/macrophage morphology after diffuse TBI in the mouse. Treatment with the IL-1β neutralizing antibody led to an attenuated loss of mature OLs at two and seven dpi without influencing OPC proliferation. Microglia/macrophage immunoreactivity was increased in brain-injured animals at two dpi, which was attenuated by the IL-1β neutralizing treatment. With time, the brain-injured groups and the sham IL-1β group showed a more active microglial/macrophage morphology compared with the sham CsA group without an increased microglia/macrophage immunoreactivity. These findings might be related to the functional outcome in TBI, because an identical treatment regime normalized complex behavioral impairments and attenuated memory deficits after cFPI in mice.^29^

The inflammatory response is an important part of the secondary injury cascade after TBI. Chronic white matter inflammation has been observed post-human TBI^38,39^ as well as in several TBI models.^20,40–42^ Immune cells infiltrated from the bloodstream are rare in the injured brain after the first post-injury weeks,^43^ whereas chronic activation of microglia, macrophages, and astrocytes as well as increased levels of cytokines and chemokines can be detected months and years after injury.^44^ By normalizing pro-inflammatory cytokine levels in mice subjected to closed head injury, reduced cognitive deficits and microglial activation were detected.^45^

The cytokine IL-1β is increased after TBI and is one of the first inflammatory mediators, secreted primarily by microglia and macrophages.^20,46,47^ IL-1β is involved in apoptosis, blood–brain barrier breakdown, recruitment of other immune cells as well as in the production of pro-inflammatory mediators.^48^ In addition, IL-1β and microglia are involved in several apoptotic pathways^49–51^ and could promote OL death via glutamate excitotoxicity.^52,53^ Several studies have used therapeutic interventions to target IL-1β after TBI,^23,25,26,52,54^ where beneficial consequences on brain tissue loss, cognitive outcome, and reduction of the inflammatory response were observed. In addition, pharmacological inhibition of the post-injury rise in IL-1β levels after cFPI was achieved by the small molecule MW151.^55^

The mechanisms behind these behavioral improvements are poorly understood, and we hypothesized that neutralization of IL-1β, known to limit the post-injury inflammatory response, would enhance the survival of the OL population and promote OPC proliferation by altering the microglial/macrophage response to TBI. OL cell loss was prevented previously in a focal cortical contusion TBI model in the rat using a broad anti-inflammatory treatment.^52^ Earlier studies performed by our group showed that the IL-1β neutralizing antibody reached brain tissue in levels well above therapeutic concentrations (>0.2 μg/mL neutralize IL-1β *in vivo*) in cFPI mice, and the antibody was also detected in sham-injured mice,^30^ although in lower concentrations. This is an important premise for the present study.

Cell death is a common consequence of TBI. Cleaved capase-3 is a mediator for apoptosis,^56^ and its expression is increased in several TBI models.^57–60^ Increased cleaved caspase-3 staining was seen in the corpus callosum of brain-injured groups, which was attenuated by IL-1β neutralization at seven dpi. The signaling pathways between IL-1β and cleaved caspase-3 expression were not evaluated in the present study; however, treatment with an IL-1 receptor antagonist reduced the expression of cleaved caspase-3 in experimental spinal cord injury.^61^ It is plausible that the broad anti-inflammatory effects of the antibody treatment used here influenced other cell types, including in the vasculature,^62^ which should be addressed in future studies. In the present study, cleaved caspase-3 protein was found in close relation to CC1 positive OLs and was found to co-localize with the mature OL marker MOG at the RNA level. Co-localization of CC1 and cleaved caspase-3 could not be achieved by immunohistochemical analysis^10,11^ because the CC1 marker was frequently not detectable, plausibly because of clearance or degradation. Instead, OL quantification was made.

OLs are vulnerable to several of the molecular events occurring post-TBI^63^ and dead/dying OLs were detected after human and experimental TBI.^10–12^ In our present study, a reduced number of CC1 positive OLs were observed in the corpus callosum and external capsule of brain-injured controls. The TBI-induced oligodendrocyte loss was attenuated by IL-1β neutralization, particularly in parts of the external capsule affected by the transmitted pressure pulse generated in the cFPI model.^64,65^

The role of the inflammatory response on OPC is dual, where some inflammatory signals may promote whereas others inhibit their recruitment, proliferation, and differentiation. Primarily, IL-1β is believed to indirectly stimulate immune cells as well as astrocytes to produce growth factors and inflammatory signals promoting or inhibiting OPC proliferation and differentiation.^66^ OLs and OPCs, however, also express the receptor for IL-1β (type 1 interleukin receptor; IL-1r) and produce IL-1β endogenously.^67^ IL-1β itself is normally involved in signal transduction pathways of OPCs and OLs, and the role of IL-1β may be different in injured OLs and OPCs than in the healthy brain.^9,67^ OPC proliferation increases in several TBI models.^13–15,68^

Using the cFPI model in mice, we showed that the OPC proliferation peaks at seven dpi in important white matter tracts.^69^ These results were corroborated by the present study. Neutralization of IL-1β did not influence the proliferation of OPCs in brain-injured mice, investigated both at the protein and RNA level. In a multiple sclerosis model producing OL death, IL-1β ^-/-^ mice expressed OPCs up to six weeks after chemical depilation,^46^ also indicating that IL-1β elimination did not influence OPC proliferation. Further, IL-1β did not influence OPC proliferation in an *in vitro* study.^70^ IL-1β is not the sole inflammatory mediator influencing OPC proliferation, which may explain the unaltered proliferative response of OPCs in the cFPI model in mice and the maturation of OPCs to mature OL after TBI will be addressed in future cFPI studies.

To investigate whether IL-1β neutralization influences microglial/macrophage activation, their immunoreactivity and ramification were analyzed at two, seven, and 14 days post-injury. Ramified microglia/macrophages are considered to be in a resting state and amoeboid cells to be reactive. Ramified cells have thinner and longer processes and an increased number of intersections of the processes. Amoeboid microglia/macrophages have shorter and thicker processes and a rounded cell body, filled with engulfed debris.^71^ We previously evaluated the inflammatory response in the cFPI model and found a transient infiltration of blood-born cells into the injured brain.^30^ Thus, persistent macrophages derived from the bloodstream may have contributed to our results and the present methods cannot distinguish between blood-born macrophages and locally activated microglia.

The treatment antibody reaches the injured brain in sufficient concentration to neutralize IL-1β after cFPI, suggesting a transient capacity to cross the blood–brain barrier.^29^ Thus, although the beneficial effects observed by the treatment antibody could also be caused by peripheral effects on monocytes, the high concentration of the antibody in the injured brain could influence the local microglia response. At two dpi, quantification of microglia/macrophage (Iba1) immunoreactivity showed that neutralization of IL-1β in brain-injured animals maintains microglial processes in a similar state as in sham-injured animals—i.e., more ramified and resting.

Why the microglial/macrophage response declined beyond two days was not evaluated but might be because of a gradual decline of the neuroinflammatory response at later time points after the injury.^72^ In contrast, brain-injured controls showed microglia/macrophages in a more reactive form. Sholl analysis showed brain-injured animals and sham IL-1β animals to be in a more active form at later time points. This phenomenon has also been described after treatment with an antagonist to the interleukin 1 receptor.^73^ Here, in human TBI, the anti- IL-1β treatment caused activation of microglia with a bias toward the anti-inflammatory M2 phenotype. The phenotype of microglia was not determined in our present study and should be investigated further.

Pro-inflammatory cytokines including IL-1β may not lead to an increased number of microglial cells.^74^ In addition, when compared with the oligodendrocyte changes, the microglia/macrophages responded with a different temporal profile plausibly because of e.g., their different function and pattern of surface receptors.^75^ A broad anti-inflammatory treatment using minocycline and N-acetylcysteine also resulted in increased M1 and M2 activation after mild TBI in the rat.^52^ In a previous cFPI study, no microglial changes were observed despite lowered IL-1β levels.^55^ Only cortical changes were analyzed at an acute, 6 h, post-injury time-point, however, in contrast to the present study where the delayed response in the white matter was addressed.

Some effect of the IL-1β neutralizing treatment was observed also in sham-injured animals at 14 dpi. Previously, we showed beneficial behavioral effects of the IL-1β neutralization in sham-injured compared with naïve mice.^29^ It is possible that the neutralizing IL-1β treatment has additional mechanisms of action in sham-injured animals. IL-1β signaling plays a role not only in the diseased brain but also in normal, healthy metabolism and on cognitive function.^67,76–78^ Thus, IL-1β signaling may have beneficial effects especially when released in modest concentrations.^79^

Sham injury also evokes a minor inflammatory response,^80^ and the strength of the inflammatory input is known to influence endogenous IL-1β release. The activated microglia/macrophages seen in the sham IL-1β group was not related to infection because no late infiltration of neutrophils was found in this group. IL-1β neutralized sham-injured controls also displayed a loss of mature OLs at 14 dpi. IL-1β signaling is required for CTNF (ciliary neurotrophic factor) production important for maintaining oligodendrocyte survival^43^ as well as signaling cascades reducing oxidative stress,^81^ known to be harmful to OLs.

This study is not without its limitations. We did not determine the endogenous levels of IL-1β levels after injury and antibody treatment. Using identical dosing as in the present study, however, the brain tissue concentrations of the antibody were well above therapeutic levels at both 24 and 72 h post-injury in the cFPI model.^29^ There is also some variability of the injury response in the cFPI model, plausibly because of minor alterations of the fluid percussion pulse. This variability is reflected in the morphology of the brains as well as in the cell counting analysis. Further, stereology, the gold standard of cell counting, was not used in our study. In contrast, we used extensive and exhaustive cell counting on several sections from different bregma levels and in several vulnerable white matter tracts. Thus, we argue that our data provide a good estimate of the OL and OPC population.

## Conclusion

Our data suggest that IL-1β is negatively involved in OL cell death although not in OPC proliferation after diffuse TBI. It is plausible that the reduced OL cell death and altered microglial/macrophage morphology lead to preserved white matter integrity, contributing to the normalized behavioral profiles observed after IL-1β neutralization in the central fluid percussion model of diffuse TBI.

## Supplementary Material

Supplemental data

Supplemental data

Supplemental data
