# Supplementary material for: Neutralization of Interleukin-1β following Diffuse Traumatic Brain Injury in the Mouse Attenuates the Loss of Mature Oligodendrocytes
Source: J Neurotrauma. 2018 Nov 12;35(23):2837–49. doi: 10.1089/neu.2018.5660 (PMC6247990; doi:10.1089/neu.2018.5660)
Supplement: Supplemental data [file Supp_Fig1.pdf]

Supplementary Data

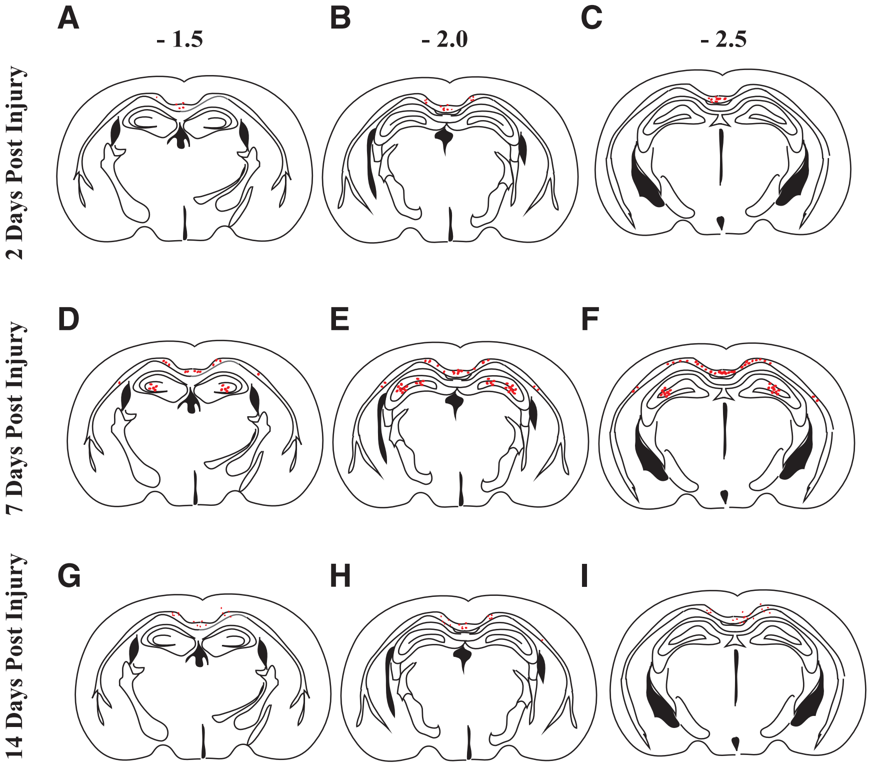

SUPPLEMENTARY FIG. S1. Schematic illustration of cleaved caspase-3 distribution (red) in cFPI CsA animals. Cleaved caspase-3 staining was made from coronal sections of three different levels from bregma (−1.5, −2.0, and −2.5). At two dpi, cleaved caspase-3 expression was similar among all treatment groups at all levels (**A–C**). At seven dpi, the caspase-3 expression was increased in brain-injured groups (**D–F**), although then declined and was similar among the treatment groups at 14 dpi (**G–I**). cFPI, central fluid percussion injury; dpi, days post-injury; CsA, inactive control antibody against cyclosporin A.
