# Supplementary material for: Neutralization of Interleukin-1β following Diffuse Traumatic Brain Injury in the Mouse Attenuates the Loss of Mature Oligodendrocytes
Source: J Neurotrauma. 2018 Nov 12;35(23):2837–49. doi: 10.1089/neu.2018.5660 (PMC6247990; doi:10.1089/neu.2018.5660)
Supplement: Supplemental data [file Supp_Fig2.pdf]

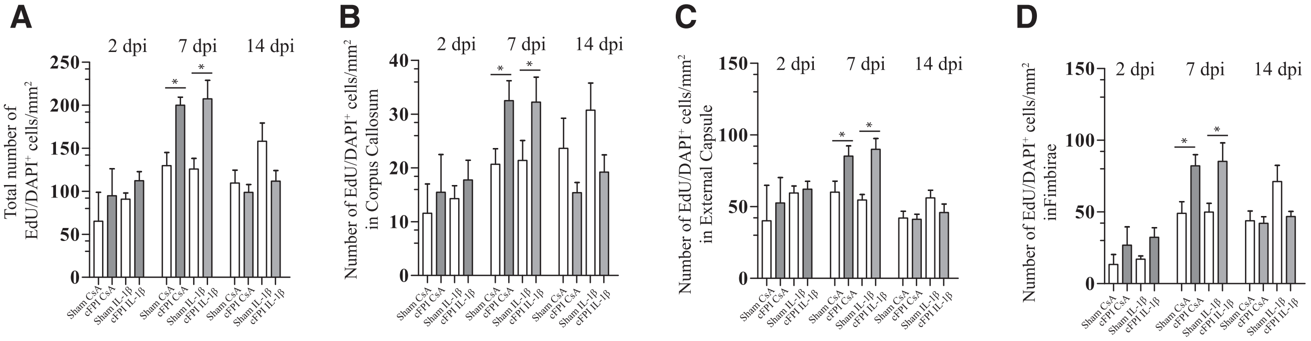

SUPPLEMENTARY FIG. S2. The number of proliferating cells in the corpus callosum, external capsule, and fimbriae. An increase (*$p \leq 0.05$) was observed in the number of proliferating cells only at seven days post-injury (dpi) group for the total number of cells (**A**), in the corpus callosum (**B**), external capsule (**C**), and fimbriae (**D**) between the brain-injured (cFPI CsA and cFPI IL-1*β*) and sham-injured (sham CsA and sham IL-1*β*) groups, although not at two or 14 dpi. In graphs, results are presented as mean and standard error of the mean. cFPI, central fluid percussion injury; dpi, days post-injury; CsA, inactive control antibody against cyclosporin A; IL-1*β*, interleukin-1 beta.
