# Supplementary material for: Neutralization of Interleukin-1β following Diffuse Traumatic Brain Injury in the Mouse Attenuates the Loss of Mature Oligodendrocytes
Source: J Neurotrauma. 2018 Nov 12;35(23):2837–49. doi: 10.1089/neu.2018.5660 (PMC6247990; doi:10.1089/neu.2018.5660)
Supplement: Supplemental data [file Supp_Fig3.pdf]

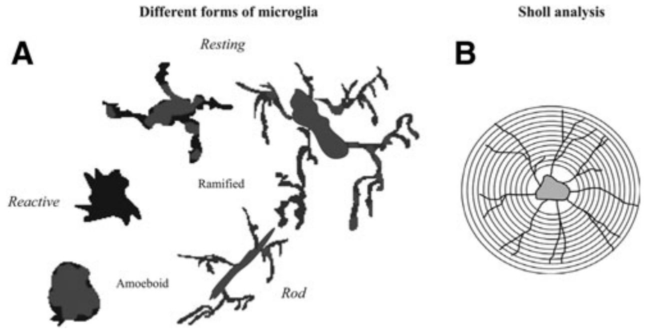

SUPPLEMENTARY FIG. S3. (**A**) Schematic illustration of microglia in different forms from resting to reactive. (**B**) Schematic illustration of the Sholl analysis of microglia/macrophages used in the present study.
